# P-1668. Association of Memory Function with COVID-19 Outcomes in Adults Aged 50 Years and Older: Analysis of Three Prospective Cohorts

**DOI:** 10.1093/ofid/ofaf695.1842

**Published:** 2026-01-11

**Authors:** Juanjuan Shi, Xin Shen, Yan Tian, Rui Lu, Xiaoli Jia, Jia Li, Xiaozhen Geng, Song Zhai, Fanpu Ji, Shuangsuo Dang, Wenjun Wang

**Affiliations:** Second Hospital of Xi'an Jiaotong University, Xi'an, Shaanxi, China; Shaanxi Provincial people’s Hospital, Xi'an, Shaanxi, China; Second Hospital of Xi'an Jiaotong University, Xi'an, Shaanxi, China; Second Hospital of Xi'an Jiaotong University, Xi'an, Shaanxi, China; Second Hospital of Xi'an Jiaotong University, Xi'an, Shaanxi, China; Second Hospital of Xi'an Jiaotong University, Xi'an, Shaanxi, China; Second Hospital of Xi'an Jiaotong University, Xi'an, Shaanxi, China; Second Hospital of Xi'an Jiaotong University, Xi'an, Shaanxi, China; Second Hospital of Xi'an Jiaotong University, Xi'an, Shaanxi, China; Second Hospital of Xi'an Jiaotong University, Xi'an, Shaanxi, China; Second Hospital of Xi'an Jiaotong University, Xi'an, Shaanxi, China

## Abstract

**Background:**

Patients with Alzheimer's disease or dementia are at increased risk for COVID-19 hospitalization and mortality. However, no study has examined whether memory function is associated with COVID-19 outcomes in general older adults.

**Figure:**
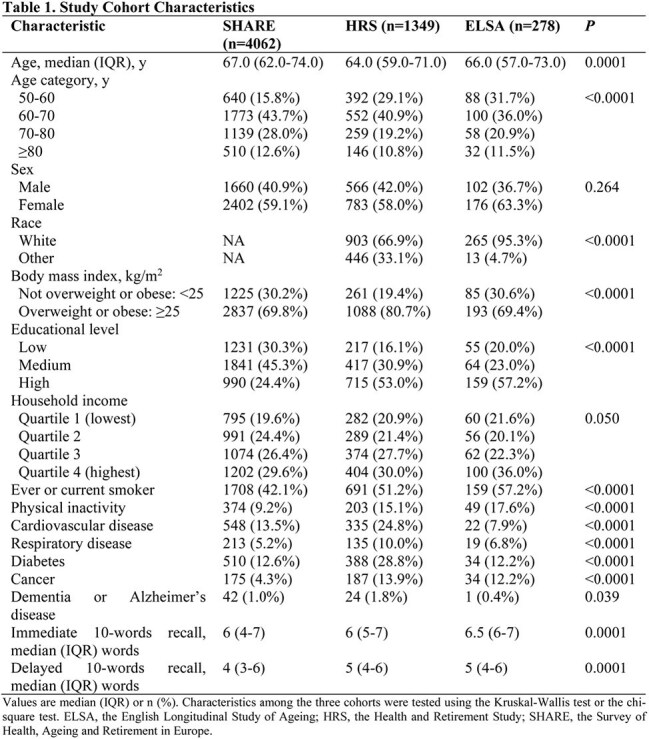

**Figure:**
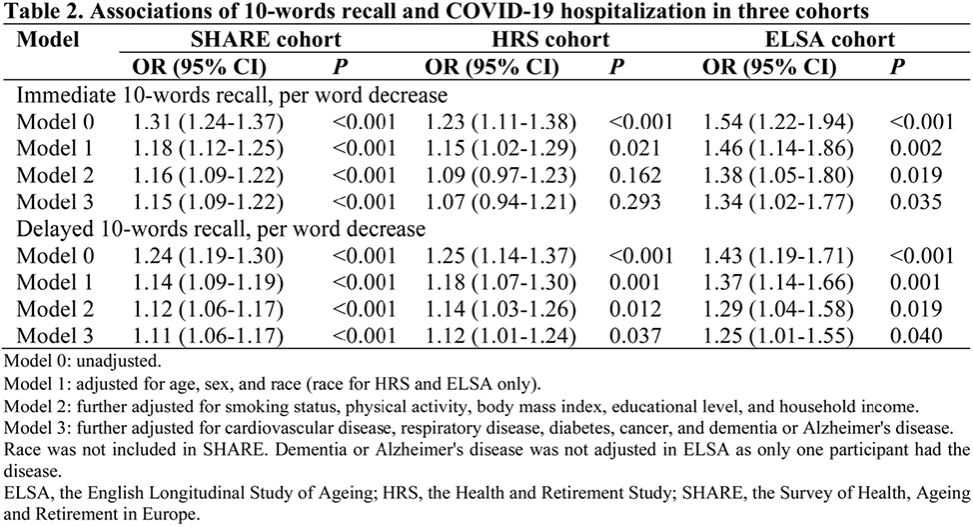

**Methods:**

Data were obtained from SHARE (the Survey of Health, Ageing and Retirement in Europe), HRS (the Health and Retirement Study), and ELSA (the English Longitudinal Study of Ageing), three prospective and representative cohorts of non-institutionalized adults aged 50 years and older in 25 European countries plus Israel, the United States, and the United Kingdom, respectively. Memory function was measured with immediate and delayed 10-words recall tests. Associations of 10-words recall with COVID-19 hospitalization and mortality were assessed using logistic models adjusted for age, sex, race, body mass index, smoking, physical activity, household income, education level, and chronic conditions.

**Figure:**
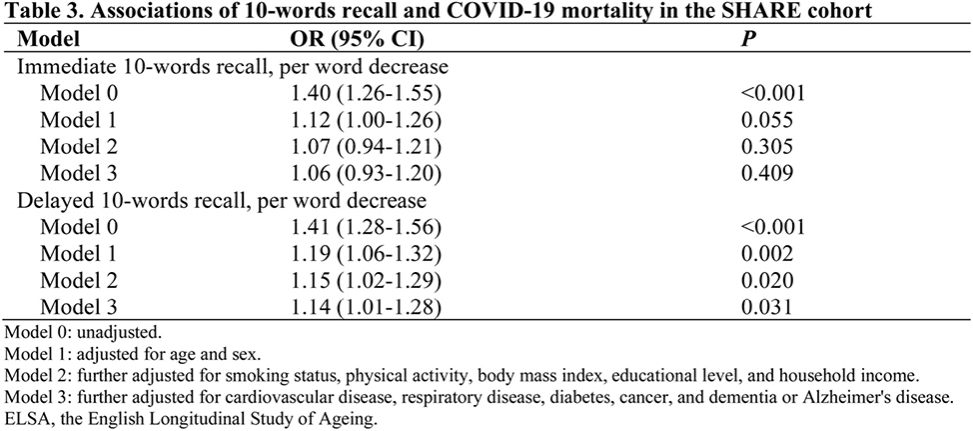

**Figure 1.**
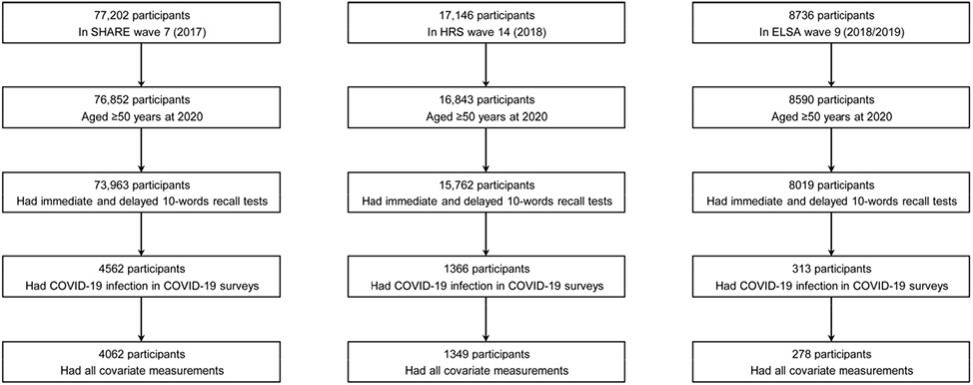
Determination of the study sample. COVID-19, coronavirus disease 2019; ELSA, the English Longitudinal Study of Ageing; HRS, the Health and Retirement Study; SHARE, the Survey of Health, Ageing and Retirement in Europe.

**Results:**

A total of 4062 participants with COVID-19 infection from SHARE, 1349 from HRS, and 278 from ELSA were included in the analysis. 610 (15.0%) in SHARE, 142 (10.5%) in HRS, and 39 (14.0%) in ELSA were hospitalized, and 102 (2.5%) died of COVID-19 or related complications in SHARE. The adjusted odds ratios (aORs) for COVID-19 hospitalization were 1.15 (95% CI, 1.09-1.22) in SHARE, 1.07 (95% CI, 0.94-1.21) in HRS, and 1.34 (95% CI, 1.02-1.77) in ELSA, per word decrease in immediate 10-words recall. For delayed 10-words recall, the corresponding aORs were 1.11 (95% CI, 1.06-1.17), 1.12 (95% CI, 1.01-1.24), and 1.25 (95% CI, 1.01-1.55), respectively. The aORs for COVID-19 mortality were 1.07 (95% CI, 0.94-1.21) and 1.14 (95% CI, 1.01-1.28) per word decrease in immediate and delayed 10-words recall in SHARE, respectively. Results were relatively robust to missing data of covariates, exclusion of cases based on symptoms alone, or exclusion of cases with Alzheimer's disease or dementia.

**Conclusion:**

This study shows that low memory performance, as measured by 10-words recall, is independently associated with an increased risk of COVID-19 hospitalization and mortality in adults aged 50 years and older.

**Disclosures:**

All Authors: No reported disclosures

